# An age‐period‐cohort approach to studying long‐term trends in obesity and overweight in England (1992–2019)

**DOI:** 10.1002/oby.23657

**Published:** 2023-02-06

**Authors:** Magdalena Opazo Breton, Laura A. Gray

**Affiliations:** ^1^ School of Health and Related Research University of Sheffield Sheffield UK; ^2^ Healthy Lifespan Institute University of Sheffield Sheffield UK

## Abstract

**Objective:**

This study aims to understand long‐term trends in obesity and overweight in England by estimating life‐course transitions as well as historical and birth cohort trends for both children and adults.

**Methods:**

Data on individuals aged 5 to 85 years old from the Health Survey for England were used, covering the period 1992 to 2019 and birth cohorts born between 1909 and 2013. Individual BMI values were classified as healthy weight, overweight, or obesity. Trends were compared, and an age‐period‐cohort model was estimated using logistic regression and categorical age, period, and cohort groups.

**Results:**

There was significant variation in age trajectories by birth cohorts for healthy weight and obesity prevalence. The odds of having obesity compared with a healthy weight increased consistently with age, increased throughout the study period (but faster between 1992 and 2001), and were higher for birth cohorts born between 1989 and 2008. The odds of having overweight showed an inverted U‐shape among children, increased through adulthood, have been stable since 2012, and were considerably higher for the youngest birth cohort (2009–2013).

**Conclusions:**

Younger generations with higher overweight prevalence coupled with increasing obesity prevalence with age suggest that obesity should remain a high priority for public health policy makers in England.

**Study Importance**

**What is already known?**

- The risk of living with obesity increases throughout adulthood, with birth cohorts aged above 18 years old having a considerably higher risk than younger birth cohorts and with birth cohorts born after the 1960s at higher risk than their predecessors.

**What does this study add?**

- The risk of living with obesity has not increased linearly with time but experienced a faster increase between 1992 and 2001, and it has been especially high for birth cohorts between 1989 and 2008.
- The risk of having overweight shows an inverted U‐shape among children and an increasing trend during adulthood; it has remained fairly the same through time but it has been considerably higher among those born around and after the 1990s and especially high for those born between 2009 and 2013.

**How might these results change the direction of research or the focus of clinical practice?**

- The risk of having overweight has remained fairly similar in England since 1992, but the risk of obesity has increased considerably. Both overweight and obesity have a clear increasing age pattern among adults, but the risk of having overweight has an inverted U‐shape in children, suggesting that there is space for interventions at ages 16 to 17 years old to stop children from transitioning into obesity when adults, in the context of significantly higher prevalence of overweight in younger birth cohorts.

## INTRODUCTION

Obesity and overweight are increasingly prevalent across Western societies. Official statistics for England for the period 1993 to 2019 show that the prevalence of obesity increased from 15% to 28%, while the prevalence of overweight and obesity combined increased from 54% to 64% [1]. Although there was evidence of obesity and overweight in children leveling off around 2004 [2], figures from the National Child Measurement Programme showed that, during the COVID‐19 pandemic, childhood obesity experienced the highest increase in England over the past 15 years [3], and it has continued to remain higher than before the pandemic [4]. Comparisons across European countries show that England had one of the highest prevalences of both obesity and overweight in 2010 among the adult population [5]. This constitutes a major risk for adults owing to the association between obesity/overweight and serious chronic conditions such as high blood pressure and high cholesterol, type 2 diabetes, breathing problems [6], consequent risk of cancer, premature death, and low quality of life, whereas for children, it is associated with psychological problems, low self‐esteem, social problems [6], and earlier development of noncommunicable diseases such as diabetes and cardiovascular diseases [7].

Age‐period‐cohort (APC) models have previously been used to model several public health topics such as smoking [8, 9, 10], alcohol use [11, 12], suicide [13, 14], drug use [15, 16], mental health [17], obesity [18, 19, 20, 21, 22, 23, 24, 25], and overweight [26]. Their purpose is to disentangle the effects of age (life cycle), time (historical), and generation associated with risky behaviors and disease, allowing researchers to better understand long‐term trends [8] and to predict future scenarios [27], as well as to provide vital information for policy makers and public health surveillance [28].

Following a similar APC approach used to understand long‐term trends in alcohol [11] and smoking [8], we aimed to present evidence and contribute to the understanding of life cycle and historical and generational effects of the long‐term prevalence of overweight and obesity in England during 28 years, for both children and adults and birth cohorts born between 1909 and 2013.

## METHODS

### Data sources

We used yearly, cross‐sectional, individual‐level data from a population aged 5 to 85 years old using the Health Survey for England (HSE) for the period 1992 to 2019 (excluding boost samples used across the years [29]). The HSE is a continuous survey designed to monitor the population's health and care annually, and it collects information from individuals living in private households in England.

### Outcome variable

We obtained measures of body mass index (BMI) from yearly HSE surveys following Higgins and Marshall [30]. BMI was calculated using valid weight in kilograms and valid height in meters (survey valid measures refer to “no problems were experienced while taking the measures”). We categorized BMI into one of three categories: healthy weight (18.5 kg/m^2^ ≤ BMI < 25 kg/m^2^), overweight (25 ≤ BMI < 30), and obesity (BMI ≥ 30). Measures for children were extrapolated from adults' BMI classification based on the International Obesity Taskforce definitions and using values for males and females separately [31, 32]. Adults and children classified as underweight (BMI < 18.5 or childhood equivalent [31, 32]) were not included in our analysis. Using weight status, we created two dummy variables for the APC analysis: (a) to compare obesity with a healthy weight and (b) to compare overweight with a healthy weight.

### APC variables

The HSE contains single years of age from 1992 to 2014 and only categorical age for the period 2015 to 2019. We imputed single years of age for all values that had only categorical age using a multiple imputation model (more details in online Supporting Information). To deal with differences in terms of BMI classification between children and adults, we grouped by age to display general population prevalence trends for children (5–17 years old) and adults (18–85 years old) and used 18 years old as a reference category in our APC estimation. We grouped survey years into 14 groups of 2‐year periods (from 1992–1993 to 2018–2019) and grouped year of birth (computed using age and survey year or imputed age and survey year) into 21 groups of 5‐year birth cohorts, as is frequently done in APC models [33]. The 5‐year birth cohort groups ranged from 1909–1913 to 2009–2013, and all had sample sizes above 1000 observations (Supporting Information Table S1).

### APC models

APC models are characterized by an intrinsic “identification problem” associated with the linear dependency between age (A), period (P), and birth cohort (C), because *C = P − A* [34]. There are many different solutions to deal with the perfect multicollinearity between the three variables [35], with one of them being to transform each of the three variables and create age groups, period groups, and birth cohort groups of different sizes to break the strict dependence among the variables. Following this approach, we created 2‐year survey periods and 5‐year birth cohorts, under the assumption that the groups created were internally homogeneous and externally heterogeneous enough to allow finding age, period, and cohort effects. We used single years of age because the definition of weight status differed before and after age 18 years, making it harder to assume any groupings. Therefore, our strategy to deal with the identification problem included visually displaying the age trajectories for each of our BMI categories to find APC patterns in the data [35]; estimating three APC models using grouped period and birth cohort variables but single years of age; and then running a series of sensitivity analyses using different groupings for age, period, and birth cohort in order to check the robustness of our results.

### Statistical analysis

We first plotted prevalence for each of the three BMI categories (healthy weight, overweight, and obesity) by survey year and children and adults separately. Next, with our imputed age variable and survey year, we computed our birth cohort variable and graphically displayed each of the outcome variables over age, by 5‐year birth cohort, and finally, we estimated an APC model. We used logistic regressions for each of our binary outcome variables following a similar model used for smoking [8] and alcohol [11] in order to study obesity and overweight in the wider context of public health topics. We ran two regression models, one for each outcome variable, using categorical single years of age (reference category: 18 years old), 2‐year survey period (reference category: 1992–1993), and 5‐year birth cohort (reference category: 1909–1913). Results were displayed graphically in terms of odds ratio (OR) and their 95% confidence intervals (95% CI). We performed three sensitivity analyses to check the robustness of our APC estimation using different groupings for age, period, and birth cohort. All analyses was done in Stata version 17 (StataCorp). We did not include sample weights because they were not available for the whole study period, but the evidence suggests there is a good match between HSE and the population it refers to [36].

## RESULTS

### Prevalence of healthy weight, overweight, and obesity

**FIGURE 1:**
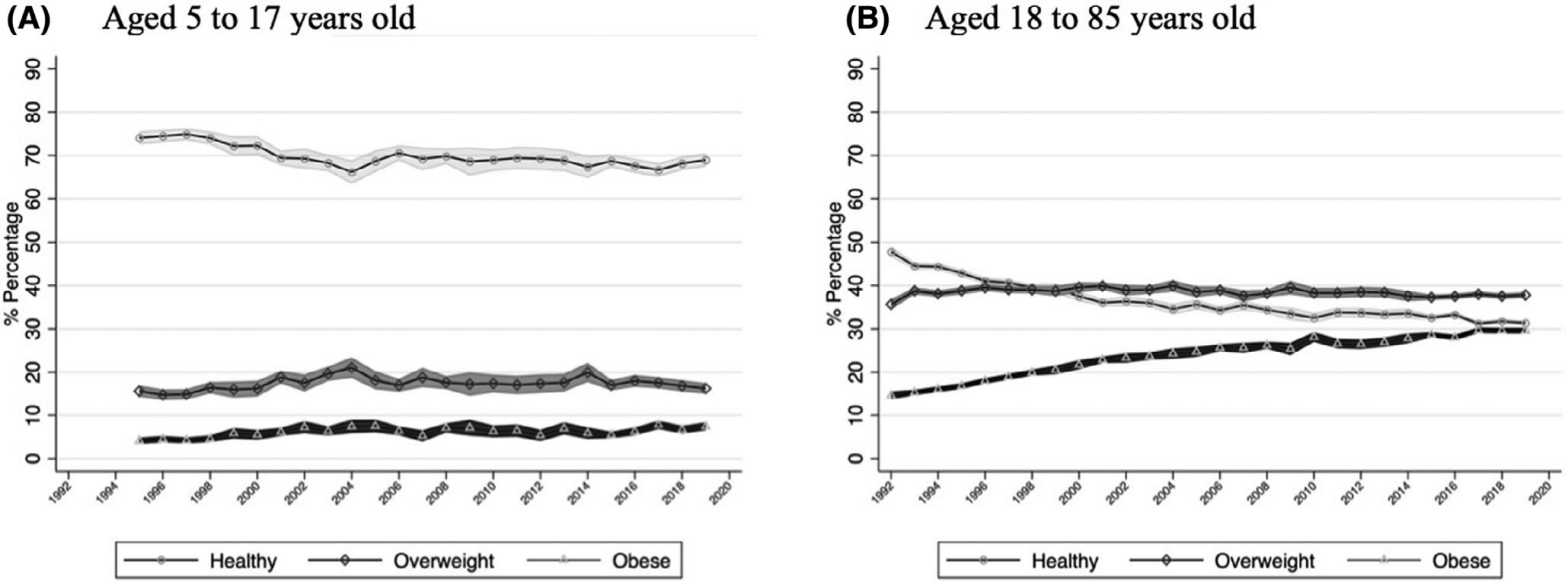
Prevalence of healthy weight, overweight, and obesity in England by year and age group: (A) children aged 5 to 17 years old (Health Survey for England, 1995–2019); (B) adults aged 18 to 85 years old (Health Survey for England, 1992–2019)

Our sample consisted of 377,696 individuals with valid BMI measures (Supporting Information Figure S1 shows that we had valid measures of BMI for more than 80% of the sample for all survey years and no clear pattern in missing data in relation to available sociodemographic variables). In Figure 1A, children (aged 5 to 17 years old) showed a decrease in healthy weight prevalence during the period studied from 74% in 1995 (95% CI: 73%–76%) to 69% in 2019 (95% CI: 67%–71%) and an increase in obesity from 4% in 1995 (95% CI: 3%–5%) to 8% in 2019 (95% CI: 7%–8%), whereas overweight remained relatively stable at around 16% for the whole study period. In Figure 1B, adults (aged 18 to 85 years old) showed a marked decrease in healthy weight from 48% in 1992 (95% CI: 47%–49%) to 31% in 2019 (95% CI: 31%–32%) and a marked increase in obesity prevalence from 15% in 1992 (95% CI: 14%–16%) to 30% in 2019 (95% CI: 29%–30%) during the period studied but only a slight increase in overweight prevalence from 36% in 1992 (95% CI: 35%–37%) to 38% in 2019 (95% CI: 37%–39%). Therefore, our figures suggested that, by 2019, the percentage of adults living with a healthy weight was almost the same as those who were living with obesity.

### Age trajectories by 5‐year birth cohorts

We observed birth cohorts for between 6 and 28 survey years, with the longest series obtained for birth cohorts born between 1924 and 1978 and the shortest for birth cohorts born between 2009 and 2013. Of the 21 birth cohorts, 13 had 25 or more years of survey data. Birth cohorts born between 1999 and 2013 were observed mainly while aged less than than 18 years old.

**FIGURE 2:**
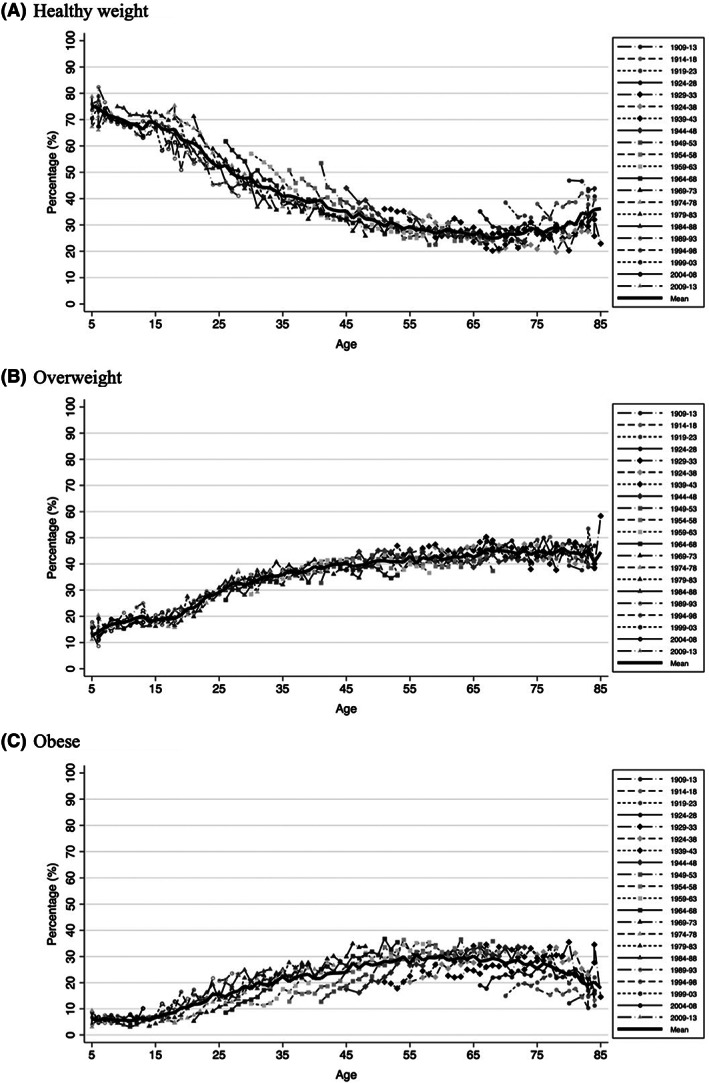
Age trajectories in healthy weight, overweight, and obesity by age for each 5‐year birth cohort in England using Health Survey for England, 1992–2019

Figure 2 showed that there was a marked decrease in the prevalence of healthy weight for all birth cohorts as they aged (Figure 2A). The decrease was especially steep between the ages of 15 and 35 years old, and it continued until around the age of 65, while for the prevalence of overweight and obesity there was an increase from the age of 5 up until the age of 65 years old (Figure 2B and 2C, respectively). Interestingly, the age trajectory of overweight prevalence was very similar among birth cohorts (Figure 2B). In terms of obesity, birth cohorts born between 1944 and 1968 showed a faster increase with age in obesity prevalence compared with others. The prevalence of obesity peaked for most birth cohorts around their most recent observation, with as high as 37% of their 5‐year birth cohort population classified as living with obesity in birth cohorts 1949–1953 and 1964–1968.

### APC estimation

**FIGURE 3:**
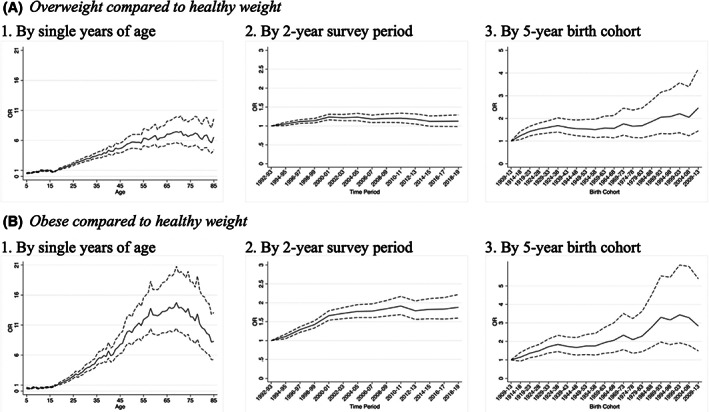
Estimated odds ratios (solid line) and 95% confidence intervals (dash lines) of having (A) overweight compared with a healthy weight and (B) obesity compared with a healthy weight by single years of age, 2‐year survey period, and 5‐year birth cohort using Health Survey for England, 1992–2019. Reference category for age is 18 years old, for period is the 1992–1993 period, and for birth cohort is the birth cohort born between 1909 and 1913.

Figure 3 graphically displays the results of our APC analysis, while full regression results can be found in Supporting Information Table S2 and S3. Period effects and age effects followed similar patterns as those observed in Figures 1 and 2, respectively. In terms of age effects, our model showed that, compared with healthy weight, the odds of both overweight and obesity increased with age, peaked at 69 years old, and then decreased. However, there was a small decrease in overweight at 16 years old (OR: 0.79; 95% CI: 0.70–0.89) and 17 years old (OR: 0.81; 95% CI: 0.72–0.92) before a subsequent increase again after age 18 years (reference group), whereas the odds of living with obesity consistently increased with age.

In terms of the period effect, the odds of living with obesity increased more through time than the odds of having overweight. When comparing obesity with healthy weight, there was a steep increase in the odds of living with obesity until 2000–2001 (OR: 1.66; 95% CI: 1.54–1.79) compared with 1992–1993, with a slower increase until 2010–2011 (OR: 1.91; 95% CI: 1.69–2.17) and a small decrease in 2012–2013 (OR: 1.79; 95% CI: 1.56–2.05). Then the odds remained relatively stable, with a small increase in 2018–2019 (OR: 1.88; 95% CI: 1.60–2.22). The odds of having overweight also increased faster until 2000–2001 but to a smaller extent (OR: 1.23; 95% CI: 1.16–1.31), remained relatively stable until 2010–2011, and slightly decreased until 2018–2019 (OR: 1.13; 95% CI: 0.98–1.29).

Our cohort effects showed that the odds of living with obesity compared with healthy weight were higher among younger birth cohorts compared with older ones, with the highest odds observed for birth cohorts 1989–1993 (OR: 3.30; 95% CI: 1.97–5.54), 1994–1998 (OR: 3.16; 95% CI: 1.83–5.47), 1999–2003 (OR: 3.44; 95% CI: 1.92–6.13), and 2004–2008 (OR: 3.29; 95% CI: 1.78–6.05). However, the odds were lower (compared with their predecessors) for the youngest birth cohort, 2009–2013 (OR: 2.83; 95% CI: 1.49–5.39), probably because we observed this birth cohort only from 5 to 8 years old, when the odds of living with obesity were low compared with later ages. The odds of having overweight compared with healthy weight slowly increased with each successive birth cohort and they were twice as high for birth cohorts born after 1989, peaking for our youngest birth cohort (OR for 2009–2013: 2.46; 95% CI: 1.45–4.19). The sensitivity analyses showed that our APC estimation results were robust to different grouping specifications (more details in online Supporting Information).

### Sensitivity analyses

Figures for the sensitivity analyses can be found in Supporting Information Figures S3–S5. Our APC estimation results were robust to different grouping specifications. Age and period effects were very similar in the three sensitivity analyses, with smoother curves when larger groupings (5‐year age groups and 5‐year survey periods) were used.

## DISCUSSION

This study uses an APC model to investigate the prevalence of overweight and obesity over time in England covering a period of 28 survey years (1992–2019), 75 years of age (5–80 years old), and 24 groups of 5‐year birth cohorts (1909–2013). Our APC analysis found that the likelihood of living with obesity increased with age but more steeply between 18 and 69 years old, displaying a skewed inverted U‐shape among the adult population; it increased throughout the entire study period but faster between 1992 and 2001, and it increased to a larger extent among those born between 1989 and 2008 compared with older birth cohorts. The likelihood of having overweight showed an inverted U‐shape among children: it decreased at the ages of 16 and 17 years old but then increased between the ages of 18 and 69 years old; it experienced a small increase until 2013 but remained fairly similar during our study period; and it increased consistently within successive birth cohorts and peaked for those born between 2009 and 2013. Therefore, increases in the risk of obesity over time and a high risk of overweight among the youngest birth cohorts pose a big threat to the health of the English population.

Our results are consistent with other APC studies on obesity in the adult population showing an inverted U‐shape age effect among the population older than 18 years old [18, 19, 21, 23, 37, 38] and increasing with age for those younger than 18 years old [22], but interestingly, we found a decrease in overweight at 16 to 17 years old. The age at which obesity peaked in our study was similar to that found in a study for Australia [18], but it differed from studies in Ireland [21], the United States [24], France [20], Estonia [19], and New Zealand [37], where the peak was at a slightly younger age. This could be because most of these studies performed an APC analysis on continuous BMI rather than categories of BMI or because they covered a period earlier than that covered in our study (most covered periods before 2010), meaning that they covered an earlier stage in terms of aging population [39] or a worsening situation in terms of obesity.

In terms of period effects, our results are consistent with increasing period effects for obesity found in countries such as Australia [18, 38], the United States [23, 24], Ireland [21], and New Zealand [37], although our results for England did not linearly increase as was found in those studies. Our period effects showed that, whereas overweight remained relatively stable or even decreased after 2001, obesity experienced a rapid increase from 1992–1993 to 2000–2001 and slower rate increases until 2010–2011, and it plateaued from 2012 until the end of the study period. During these 28 years, two national obesity strategies were published in the first period of fast increase (1992–1993 to 2000–2001), seven during the second period of a slower rate increase (2002–2003 to 2010–2011), and three during the last period of sustained obesity (2012–2019) [40]. Similarly, 10 (of 12) National Institute for Health and Care Excellence guidelines related to the direct prevention and treatment of obesity were launched between 2002 and 2011, and two extra were added after [41]. Therefore, our results indirectly suggest that these national efforts have been successful in lowering the rate at which obesity was growing, but further research is required to understand the direct causal relationship between interventions designed to tackle obesity and their effect on obesity prevalence.

Period effects in our study are promising. However, cohort effects suggest that younger cohorts have a higher likelihood of having overweight compared with their predecessors. This, along with increases in obesity with age, indicates that the obesity problem in England is still likely to be ongoing. The risk of the earlier development of noncommunicable diseases such as diabetes and cardiovascular disease is a growing public health concern.

The literature on cohort effects for obesity is mixed, with slightly negative effects in Ireland [21] and little difference among birth cohorts in Estonia [19] and in the latest US study [23]. However, increases were observed for an earlier US study [24] (although only for birth cohorts born between 1955 and 1975), for New Zealand [37] (for birth cohorts born between the 1960s and 1990s), and for Australia [18, 38] and France [20] (both for birth cohorts born after the 1960s). We found a steepening increase in the odds of obesity compared with healthy weight for cohorts born from 1960 onward; however, this is overshadowed by a much steeper increase from the late 1980s onward. This is probably because our data include both children and adults, and the existing literature is focused on the adult population. Similar to our results, the only study exploring children in the literature found a peak in obesity and overweight among the latest birth cohorts observed [22].

From a wider public health approach, our age trajectories for obesity prevalence are the opposite of what was found for smoking [8], for which younger birth cohorts had lower smoking prevalence compared with older birth cohorts. Results from APC models also show that age effects in obesity differ from tobacco and alcohol, with a clear peak in smoking likelihood at around the age of 25 years old [8] and alcohol abstention at 16 to 17 years old [11]. Period effects have been found to be decreasing for smoking [8] and increasing for alcohol abstention [11], providing evidence of a decrease in the risk of smoking and alcohol, whereas the risk of obesity has been increasing throughout a similar study period. In terms of cohort effects, the likelihood of smoking has decreased among successive birth cohorts [8] and has increased for alcohol abstention [11], whereas it has increased for obesity. Since most birth cohorts have not yet peaked in their obesity prevalence, the future seems less promising for obesity than for smoking and alcohol.

This study is the first to include both children and adults to model age, period, and cohort effects for overweight and obesity, which allowed us to extend the number of birth cohorts observed, allowed us to follow birth cohorts throughout a longer age range, and (for some birth cohorts) allowed us to follow them throughout their transition to adulthood. Our results provide a life‐course perspective on obesity and overweight, and by adding healthy weight, we explore long‐term transitions between BMI categories among successive birth cohorts. However, our results cannot directly address individual transitions, which would be possible with individual‐level longitudinal data and a different study design, since an APC analysis on obesity in Australia showed similar patterns when comparing results from cross‐sectional and longitudinal data [38]. Using a longitudinal approach to understand transitions in weight states could also contribute to a better understanding of the increases in healthy weight at older ages observed in Figure 2 and how these changes in body mass are associated with mortality in this age group [42].

Our results are also based on the use of two logistic regressions, one for overweight and one for obesity, instead of just one ordered model for the three BMI categories. Although our model choice leaves out information, it allows studying age, period, and cohort effects for obesity and overweight in the wider context of public health topics [8, 43]. However, we believe that further research is needed to understand these effects over the whole BMI spectrum.

The HSE is a series of repeated cross‐sectional data, run annually since 1992. These 28 years of survey data provide consistent measures of BMI from a representative sample of the population, allowing us to extend previous international APC studies on obesity [18, 19, 21, 24, 25, 26, 37]. However, there are limitations to these data. First, the End of User licensed HSE (available at https://ukdataservice.ac.uk/) does not include single years of age from 2015 to 2019, which meant that we had to impute it to perform our APC analysis. Although this is not ideal, we believe that the methods proposed can provide a useful tool for other researchers to use these easy‐access data sets on other important health topics without the need to apply for the secure version of the data [29] (only available until 2015) while still ensuring the anonymity of the survey participants. Second, data on children started being collected only in 1995, with up to two randomly selected children until 2000 and up to four children from 2001. From an APC perspective, information on children is mainly used to extend the birth cohorts, and we have been careful to include only estimations that use at least 100 observations. Finally, data were available until 2019, and the latest literature suggests that the situation might be different after 2020 [7]. Further research could explore inequalities in obesity and overweight, as well as the effects of the COVID‐19 pandemic.

Our study suggests that obesity prevalence has stabilized since 2012; however, a combination of increased obesity with age and a considerably higher likelihood of overweight in younger birth cohorts presents a risk to the health of the English population that is likely to continue. Future prevention policies on young people have the opportunity to focus on those aged 16 and 17 years old, whose risk of having overweight is lower and who are about to transition into adulthood, during which the risk of obesity and overweight increases almost linearly with age. Future policies would also benefit from using a birth cohort approach, that is, interventions that not only can affect an age group but have the potential to change transitions into obesity and overweight among successive generations, like the birth cohort smoking ban proposed in New Zealand [44].

## FUNDING INFORMATION

This study is funded by Medical Research Council (MRC) grant numbers MR/T032472/1 and MR/S009868/1. The views expressed are those of the authors and not necessarily those of the MRC. For the purpose of open access, the authors have applied a Creative Commons Attribution (CC BY) license to any author‐accepted manuscript version arising.

## CONFLICT OF INTEREST

The authors declared no conflict of interest.

## Supporting information


**Appendix S1.** Supporting information

## Data Availability

The data underlying this article (HSE) are available with an End of User License at the UK Data Service website (http://beta.ukdataservice.ac.uk/datacatalogue/series/series?id=2000021).
