# Supplementary material for: An age‐period‐cohort approach to studying long‐term trends in obesity and overweight in England (1992–2019)
Source: Obesity (Silver Spring). 2023 Feb 6;31(3):823–31. doi: 10.1002/oby.23657 (PMC10947422; doi:10.1002/oby.23657)
Supplement: Supplementary file 1 — Appendix S1. Supporting information [file OBY-31-823-s001.docx]

**SUPPLEMENTARY MATERIAL**

Figure S1: Percentage of cases with valid BMI for young (aged 5 to 17 years old, HSE 1995-2019) and adults (aged 18 to 85 years old, HSE 1992-2019) by year, Health Survey for England (1992-2019) [1-28]


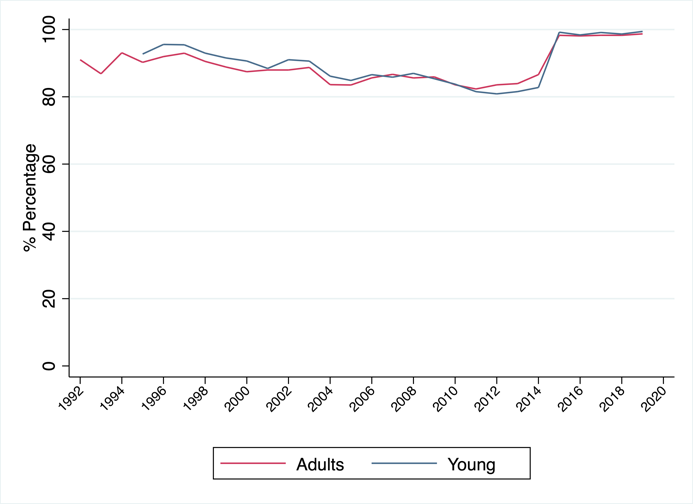


Note: A series of field quality control measures were introduced to the survey in 2015 [29]

We checked patterns of missingness between valid BMI and some of the available sociodemographic variables such as socioeconomic status of the head household, ethnicity, and region. Our data showed that 1,490 out of the 18 years old and above population (321,102) had missing values in both valid BMI and socioeconomic status of the head of household (manual/non-manual), and 1,159 in both valid BMI and ethnicity (white/other). For the population aged 5 to 15 years old (56,594), the numbers were 313 missing values in both valid BMI and socioeconomic status of the household and 170 in both valid BMI and ethnicity.

**Multiple imputation of single years of age**

We tried two imputation methods: multiple imputation and random imputation [30]. We chose multiple imputation over random imputation since the number of periods for which each birth cohort was observed and the number of observations per birth cohort was larger, allowing us to have a longer series of observations for each birth cohort and more power for the estimation (Table S1). We also checked the robustness of our multiple imputation method comparing a graphical representation of age trajectories by birth cohort (Figure S2) using data on smoking prevalence from the HSE with imputed age and data from different surveys that have single years of age [31]. Our multiple imputation model used the categorical age variable defined for the survey years 2015 to 2019 (which we extended for survey years 1992-2014) and other variables highly correlated with biological age, such as systolic and diastolic blood pressure, weight and height and controlled by sex [32, 33] to impute single years of age for all observations between 2015 and 2019.

Table S1: Sample comparison of number of periods observed, total observations and mean, minimum and maximum age by birth cohort for random imputation of age (based on categorical age variable) and multiple imputation model, Health Survey for England (1992-2019)

| Birth cohort (N) | Random imputation of age | | | | | | | | Multiple imputation of age | | | | |
| --- | --- | --- | --- | --- | --- | --- | --- | --- | --- | --- | --- | --- | --- |
|  | Number | | | Age | | | | | Number | | Age | | |
|  | Periods | Obs. | Mean | | Min | | Max | | Periods | Obs. | Mean | Min | Max |
| 1909-1913 | 7 | 1703 | | 83.1 | | 78 | | 85 | 7 | 1703 | 83.1 | 78 | 85 |
| 1914-1918 | 12 | 4148 | | 81.0 | | 73 | | 85 | 12 | 4148 | 81.0 | 73 | 85 |
| 1919-1923 | 17 | 8231 | | 78.5 | | 68 | | 85 | 17 | 8231 | 78.5 | 68 | 85 |
| 1924-1928 | 22 | 11260 | | 76.2 | | 63 | | 85 | 22 | 11260 | 76.2 | 63 | 85 |
| 1929-1933 | 23 | 14282 | | 71.9 | | 58 | | 85 | 27 | 15652 | 73.8 | 58 | 85 |
| 1934-1938 | 23 | 15698 | | 66.9 | | 53 | | 80 | 28 | 20409 | 69.4 | 53 | 85 |
| 1939-1943 | 23 | 17151 | | 61.9 | | 48 | | 75 | 28 | 24039 | 64.4 | 48 | 80 |
| 1944-1948 | 23 | 21971 | | 56.9 | | 43 | | 70 | 28 | 31882 | 59.4 | 43 | 75 |
| 1949-1953 | 23 | 21116 | | 52.0 | | 38 | | 65 | 28 | 31669 | 54.5 | 38 | 70 |
| 1954-1958 | 23 | 21317 | | 47.0 | | 33 | | 60 | 28 | 31164 | 49.5 | 33 | 65 |
| 1959-1963 | 23 | 23938 | | 42.0 | | 28 | | 55 | 28 | 33996 | 44.5 | 28 | 60 |
| 1964-1968 | 23 | 24926 | | 37.0 | | 23 | | 50 | 28 | 34925 | 39.5 | 23 | 55 |
| 1969-1973 | 23 | 21417 | | 32.1 | | 18 | | 45 | 28 | 30855 | 34.6 | 18 | 50 |
| 1974-1978 | 23 | 17040 | | 27.1 | | 16 | | 40 | 28 | 26012 | 29.6 | 16 | 45 |
| 1979-1983 | 20 | 14836 | | 23.5 | | 12 | | 35 | 25 | 23609 | 26.0 | 12 | 40 |
| 1984-1988 | 20 | 15065 | | 18.5 | | 7 | | 30 | 25 | 22703 | 21.0 | 7 | 35 |
| 1989-1993 | 20 | 14762 | | 13.6 | | 5 | | 25 | 25 | 20492 | 16.1 | 5 | 30 |
| 1994-1998 | 17 | 9463 | | 10.5 | | 5 | | 20 | 22 | 13582 | 12.9 | 5 | 25 |
| 1999-2003 | 11 | 5774 | | 8.5 | | 5 | | 15 | 16 | 11081 | 10.7 | 5 | 20 |
| 2004-2008 | 6 | 2538 | | 6.3 | | 5 | | 10 | 11 | 9613 | 8.4 | 5 | 15 |

Figure S2: Comparison of age trajectories of smoking prevalence by 5-year birth cohort: a) multiple imputation of age using Health Survey England (1992-2019), b) using different surveys to create the sample, Health Survey for England (1992-2011) and Annual Population Survey (2012-2019) based on Opazo et al (2021) [31], and c) random imputation of age using Health Survey England (1992-2019)

| a. Multiple imputation of age to create full sample |
| --- |
| 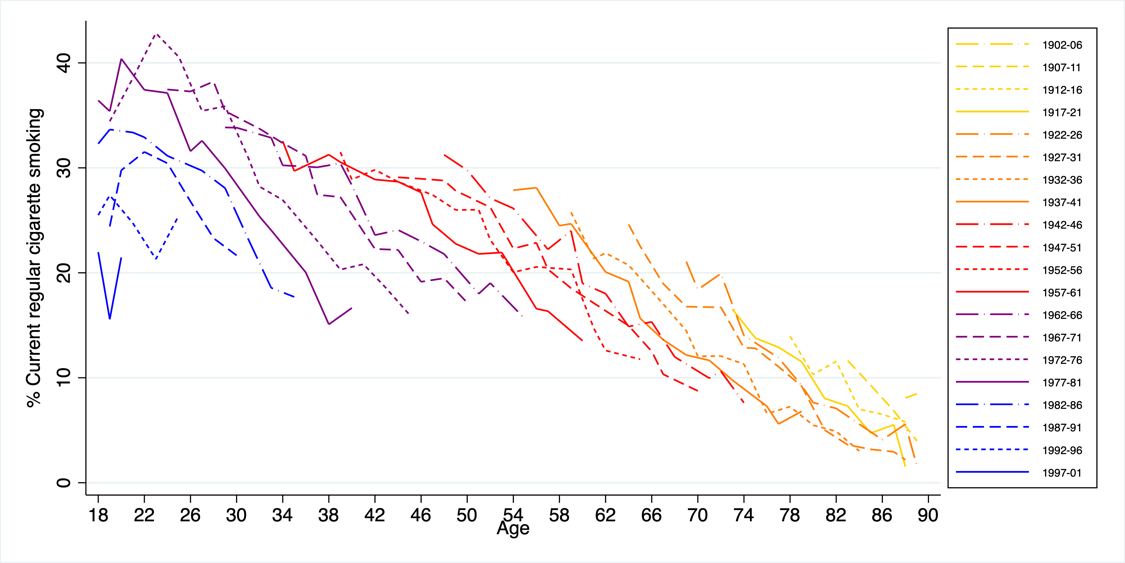 |
| b. Using different surveys to create full sample |
| 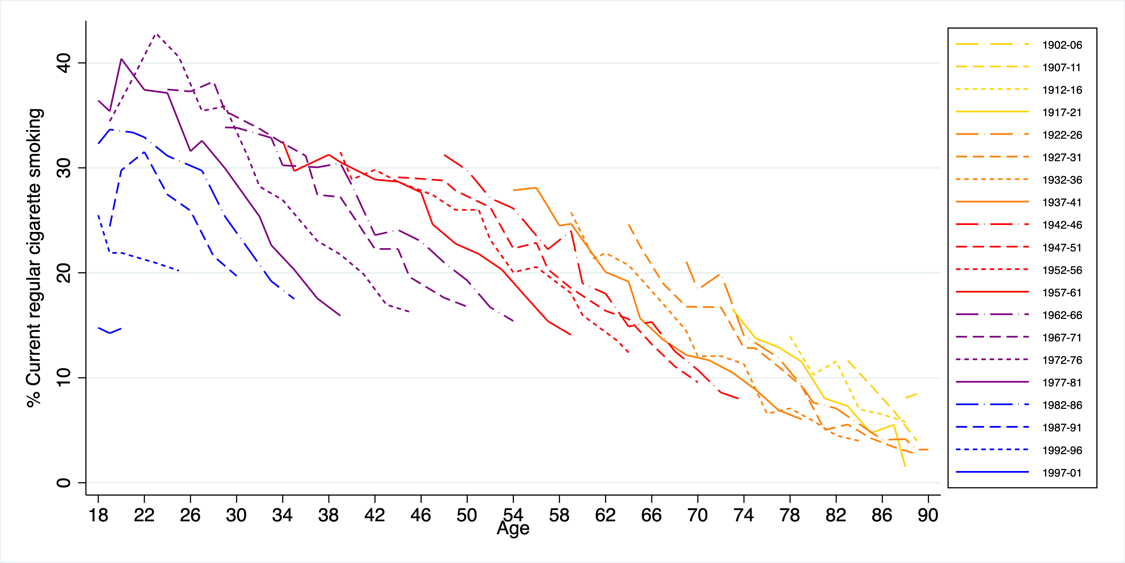 |
| c. Random imputation of age to create full sample |
| 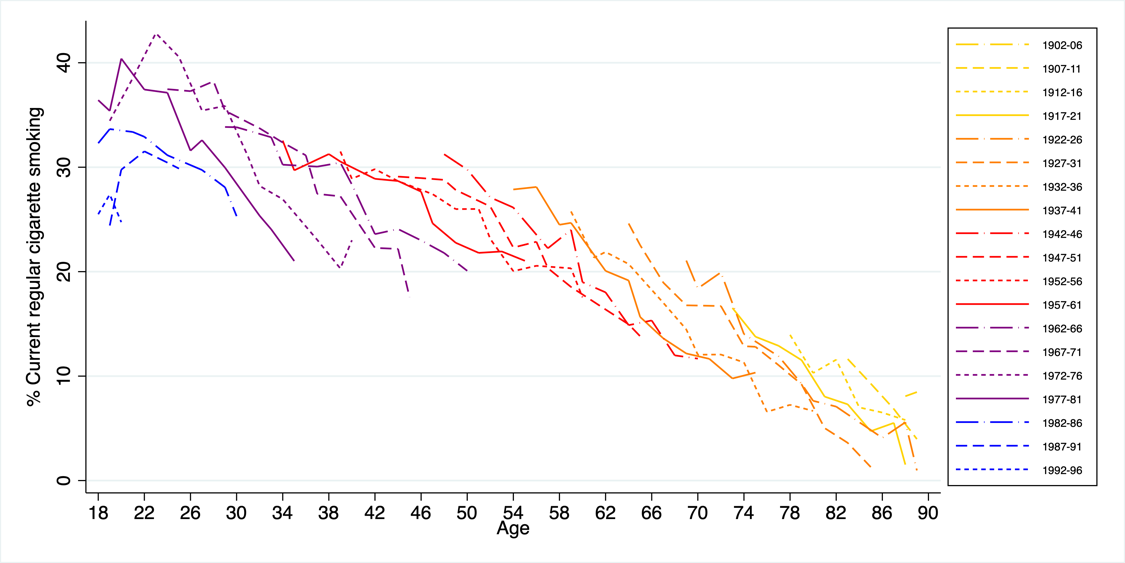 |

Table S2: APC regression results: Estimated odds (OR) of being overweight compared to healthy weight and 95% confidence interval, England (1992-2019)

|  | OR | 95% Confidence Interval | |  |  | OR | 95% Confidence Interval | |  |
| --- | --- | --- | --- | --- | --- | --- | --- | --- | --- |
| *a. Age* | | | |  | *b. Period* | | | |  |
| 5 years old | 0.54 | 0.47 | 0.63 |  | 1992-93 | 1 | 1 | 1 |  |
| 6 years old | 0.58 | 0.50 | 0.67 |  | 1994-95 | 1.05 | 1.01 | 1.10 |  |
| 7 years old | 0.56 | 0.49 | 0.64 |  | 1996-97 | 1.12 | 1.07 | 1.17 |  |
| 8 years old | 0.71 | 0.62 | 0.80 |  | 1998-99 | 1.14 | 1.08 | 1.20 |  |
| 9 years old | 0.77 | 0.68 | 0.88 |  | 2000-01 | 1.23 | 1.16 | 1.31 |  |
| 10 years old | 0.73 | 0.65 | 0.83 |  | 2002-03 | 1.22 | 1.14 | 1.31 |  |
| 11 years old | 0.98 | 0.87 | 1.11 |  | 2004-05 | 1.23 | 1.14 | 1.33 |  |
| 12 years old | 0.96 | 0.85 | 1.08 |  | 2006-07 | 1.18 | 1.09 | 1.29 |  |
| 13 years old | 0.93 | 0.83 | 1.05 |  | 2008-09 | 1.20 | 1.09 | 1.32 |  |
| 14 years old | 0.97 | 0.86 | 1.09 |  | 2010-11 | 1.20 | 1.08 | 1.34 |  |
| 15 years old | 0.97 | 0.86 | 1.09 |  | 2012-13 | 1.18 | 1.05 | 1.32 |  |
| 16 years old | 0.79 | 0.70 | 0.89 |  | 2014-15 | 1.12 | 0.99 | 1.26 |  |
| 17 years old | 0.81 | 0.72 | 0.92 |  | 2016-17 | 1.12 | 0.99 | 1.28 |  |
| 18 years old | 1 | 1 | 1 |  | 2018-19 | 1.13 | 0.98 | 1.29 |  |
| 19 years old | 1.24 | 1.10 | 1.40 |  |  |  |  |  |  |
| 20 years old | 1.35 | 1.20 | 1.52 |  | *c. Birth cohort* | | | |  |
| 21 years old | 1.46 | 1.30 | 1.64 |  | 1909-13 | 1 | 1 | 1 |  |
| 22 years old | 1.68 | 1.49 | 1.88 |  | 1914-18 | 1.24 | 1.07 | 1.44 |  |
| 23 years old | 1.64 | 1.46 | 1.84 |  | 1919-23 | 1.42 | 1.23 | 1.65 |  |
| 24 years old | 1.93 | 1.72 | 2.17 |  | 1924-28 | 1.53 | 1.31 | 1.79 |  |
| 25 years old | 2.02 | 1.80 | 2.27 |  | 1929-33 | 1.60 | 1.36 | 1.90 |  |
| 26 years old | 2.27 | 2.02 | 2.55 |  | 1924-38 | 1.68 | 1.40 | 2.02 |  |
| 27 years old | 2.34 | 2.08 | 2.63 |  | 1939-43 | 1.60 | 1.30 | 1.96 |  |
| 28 years old | 2.55 | 2.27 | 2.87 |  | 1944-48 | 1.54 | 1.23 | 1.93 |  |
| 29 years old | 2.63 | 2.33 | 2.96 |  | 1949-53 | 1.53 | 1.20 | 1.96 |  |
| 30 years old | 2.85 | 2.52 | 3.22 |  | 1954-58 | 1.51 | 1.15 | 1.96 |  |
| 31 years old | 2.95 | 2.61 | 3.35 |  | 1959-63 | 1.57 | 1.18 | 2.10 |  |
| 32 years old | 2.94 | 2.59 | 3.34 |  | 1964-68 | 1.56 | 1.14 | 2.13 |  |
| 33 years old | 3.25 | 2.86 | 3.70 |  | 1969-73 | 1.76 | 1.26 | 2.45 |  |
| 34 years old | 3.33 | 2.92 | 3.80 |  | 1974-78 | 1.66 | 1.16 | 2.37 |  |
| 35 years old | 3.47 | 3.03 | 3.97 |  | 1979-83 | 1.68 | 1.15 | 2.46 |  |
| 36 years old | 3.64 | 3.16 | 4.18 |  | 1984-88 | 1.86 | 1.24 | 2.78 |  |
| 37 years old | 3.60 | 3.12 | 4.15 |  | 1989-93 | 2.05 | 1.33 | 3.15 |  |
| 38 years old | 3.95 | 3.41 | 4.57 |  | 1994-98 | 2.08 | 1.32 | 3.27 |  |
| 39 years old | 3.86 | 3.32 | 4.48 |  | 1999-03 | 2.21 | 1.36 | 3.57 |  |
| 40 years old | 4.35 | 3.73 | 5.07 |  | 2004-08 | 2.05 | 1.23 | 3.40 |  |
| 41 years old | 4.19 | 3.58 | 4.91 |  | 2009-13 | 2.46 | 1.45 | 4.19 |  |
| 42 years old | 4.20 | 3.57 | 4.93 |  |  |  |  |  |  |
| 43 years old | 4.72 | 4.00 | 5.57 |  |  |  |  |  |  |
| 44 years old | 4.59 | 3.87 | 5.43 |  |  |  |  |  |  |
| 45 years old | 4.65 | 3.91 | 5.54 |  |  |  |  |  |  |
| 46 years old | 4.82 | 4.03 | 5.76 |  |  |  |  |  |  |
| 47 years old | 5.11 | 4.25 | 6.13 |  |  |  |  |  |  |
| 48 years old | 5.41 | 4.49 | 6.53 |  |  |  |  |  |  |
| 49 years old | 5.53 | 4.57 | 6.70 |  |  |  |  |  |  |
| 50 years old | 5.73 | 4.71 | 6.97 |  |  |  |  |  |  |
| 51 years old | 5.72 | 4.68 | 6.99 |  |  |  |  |  |  |
| 52 years old | 5.68 | 4.63 | 6.97 |  |  |  |  |  |  |
| 53 years old | 5.70 | 4.62 | 7.03 |  |  |  |  |  |  |
| 54 years old | 5.54 | 4.47 | 6.86 |  |  |  |  |  |  |
| 55 years old | 6.39 | 5.13 | 7.96 |  |  |  |  |  |  |
| 56 years old | 6.31 | 5.04 | 7.89 |  |  |  |  |  |  |
| 57 years old | 6.11 | 4.87 | 7.68 |  |  |  |  |  |  |
| 58 years old | 6.32 | 5.00 | 7.97 |  |  |  |  |  |  |
| 59 years old | 6.59 | 5.20 | 8.35 |  |  |  |  |  |  |
| 60 years old | 6.83 | 5.36 | 8.70 |  |  |  |  |  |  |
| 61 years old | 6.08 | 4.75 | 7.78 |  |  |  |  |  |  |
| 62 years old | 6.42 | 5.00 | 8.26 |  |  |  |  |  |  |
| 63 years old | 6.48 | 5.01 | 8.37 |  |  |  |  |  |  |
| 64 years old | 6.56 | 5.06 | 8.51 |  |  |  |  |  |  |
| 65 years old | 7.06 | 5.41 | 9.21 |  |  |  |  |  |  |
| 66 years old | 7.05 | 5.38 | 9.23 |  |  |  |  |  |  |
| 67 years old | 7.18 | 5.45 | 9.45 |  |  |  |  |  |  |
| 68 years old | 7.34 | 5.55 | 9.72 |  |  |  |  |  |  |
| 69 years old | 7.47 | 5.62 | 9.94 |  |  |  |  |  |  |
| 70 years old | 7.34 | 5.49 | 9.81 |  |  |  |  |  |  |
| 71 years old | 7.51 | 5.59 | 10.10 |  |  |  |  |  |  |
| 72 years old | 6.82 | 5.06 | 9.19 |  |  |  |  |  |  |
| 73 years old | 6.98 | 5.15 | 9.47 |  |  |  |  |  |  |
| 74 years old | 6.65 | 4.87 | 9.07 |  |  |  |  |  |  |
| 75 years old | 6.86 | 5.00 | 9.41 |  |  |  |  |  |  |
| 76 years old | 6.92 | 5.02 | 9.54 |  |  |  |  |  |  |
| 77 years old | 7.23 | 5.21 | 10.02 |  |  |  |  |  |  |
| 78 years old | 7.07 | 5.07 | 9.86 |  |  |  |  |  |  |
| 79 years old | 6.46 | 4.61 | 9.05 |  |  |  |  |  |  |
| 80 years old | 6.16 | 4.36 | 8.69 |  |  |  |  |  |  |
| 81 years old | 6.79 | 4.79 | 9.64 |  |  |  |  |  |  |
| 82 years old | 6.90 | 4.83 | 9.85 |  |  |  |  |  |  |
| 83 years old | 5.98 | 4.15 | 8.61 |  |  |  |  |  |  |
| 84 years old | 5.60 | 3.86 | 8.11 |  |  |  |  |  |  |
| 85 years old | 6.56 | 4.49 | 9.58 |  |  |  |  |  |  |

Note: 236,671 individual observations used in the analysis.

Table S3: APC regression results: Estimated odds (OR) of being obese compared to healthy weight and 95% confidence interval, England (1992-2019)

|  | OR | 95% Confidence Interval | |  |  | OR | 95% Confidence Interval | |  |
| --- | --- | --- | --- | --- | --- | --- | --- | --- | --- |
| *a. Age* | | | |  | *b. Period* | | | |  |
| 5 years old | 0.51 | 0.41 | 0.62 |  | 1992-93 | 1 | 1 | 1 |  |
| 6 years old | 0.44 | 0.36 | 0.54 |  | 1994-95 | 1.11 | 1.05 | 1.17 |  |
| 7 years old | 0.44 | 0.36 | 0.54 |  | 1996-97 | 1.29 | 1.22 | 1.37 |  |
| 8 years old | 0.61 | 0.51 | 0.74 |  | 1998-99 | 1.42 | 1.33 | 1.52 |  |
| 9 years old | 0.57 | 0.48 | 0.69 |  | 2000-01 | 1.66 | 1.54 | 1.79 |  |
| 10 years old | 0.42 | 0.35 | 0.51 |  | 2002-03 | 1.72 | 1.58 | 1.87 |  |
| 11 years old | 0.56 | 0.46 | 0.67 |  | 2004-05 | 1.77 | 1.61 | 1.95 |  |
| 12 years old | 0.52 | 0.44 | 0.63 |  | 2006-07 | 1.78 | 1.61 | 1.97 |  |
| 13 years old | 0.59 | 0.50 | 0.71 |  | 2008-09 | 1.84 | 1.65 | 2.06 |  |
| 14 years old | 0.64 | 0.53 | 0.76 |  | 2010-11 | 1.91 | 1.69 | 2.17 |  |
| 15 years old | 0.64 | 0.54 | 0.77 |  | 2012-13 | 1.79 | 1.56 | 2.05 |  |
| 16 years old | 0.69 | 0.58 | 0.82 |  | 2014-15 | 1.83 | 1.58 | 2.11 |  |
| 17 years old | 0.90 | 0.77 | 1.07 |  | 2016-17 | 1.83 | 1.57 | 2.14 |  |
| 18 years old | 1 | 1 | 1 |  | 2018-19 | 1.88 | 1.59 | 2.22 |  |
| 19 years old | 1.21 | 1.02 | 1.43 |  |  |  |  |  |  |
| 20 years old | 1.41 | 1.20 | 1.67 |  | *c. Birth cohort* | | | |  |
| 21 years old | 1.55 | 1.32 | 1.82 |  | 1909-13 | 1 | 1 | 1 |  |
| 22 years old | 1.78 | 1.52 | 2.09 |  | 1914-18 | 1.15 | 0.93 | 1.41 |  |
| 23 years old | 1.91 | 1.63 | 2.23 |  | 1919-23 | 1.36 | 1.11 | 1.66 |  |
| 24 years old | 2.23 | 1.90 | 2.60 |  | 1924-28 | 1.49 | 1.21 | 1.84 |  |
| 25 years old | 2.48 | 2.12 | 2.89 |  | 1929-33 | 1.71 | 1.37 | 2.13 |  |
| 26 years old | 2.85 | 2.44 | 3.33 |  | 1924-38 | 1.84 | 1.45 | 2.33 |  |
| 27 years old | 2.69 | 2.30 | 3.14 |  | 1939-43 | 1.73 | 1.34 | 2.24 |  |
| 28 years old | 2.99 | 2.55 | 3.50 |  | 1944-48 | 1.67 | 1.26 | 2.21 |  |
| 29 years old | 3.20 | 2.73 | 3.75 |  | 1949-53 | 1.75 | 1.29 | 2.37 |  |
| 30 years old | 3.67 | 3.13 | 4.31 |  | 1954-58 | 1.75 | 1.26 | 2.43 |  |
| 31 years old | 3.91 | 3.32 | 4.60 |  | 1959-63 | 1.94 | 1.36 | 2.76 |  |
| 32 years old | 4.01 | 3.40 | 4.72 |  | 1964-68 | 2.06 | 1.41 | 3.01 |  |
| 33 years old | 4.37 | 3.69 | 5.16 |  | 1969-73 | 2.34 | 1.56 | 3.51 |  |
| 34 years old | 4.49 | 3.79 | 5.33 |  | 1974-78 | 2.09 | 1.36 | 3.23 |  |
| 35 years old | 5.13 | 4.31 | 6.10 |  | 1979-83 | 2.29 | 1.44 | 3.63 |  |
| 36 years old | 5.55 | 4.64 | 6.62 |  | 1984-88 | 2.76 | 1.69 | 4.50 |  |
| 37 years old | 5.51 | 4.60 | 6.60 |  | 1989-93 | 3.30 | 1.97 | 5.54 |  |
| 38 years old | 5.94 | 4.93 | 7.15 |  | 1994-98 | 3.16 | 1.83 | 5.47 |  |
| 39 years old | 5.75 | 4.76 | 6.94 |  | 1999-03 | 3.44 | 1.92 | 6.13 |  |
| 40 years old | 6.68 | 5.50 | 8.11 |  | 2004-08 | 3.29 | 1.78 | 6.05 |  |
| 41 years old | 5.87 | 4.82 | 7.16 |  | 2009-13 | 2.83 | 1.49 | 5.39 |  |
| 42 years old | 6.12 | 5.00 | 7.49 |  |  |  |  |  |  |
| 43 years old | 6.96 | 5.66 | 8.57 |  |  |  |  |  |  |
| 44 years old | 7.28 | 5.89 | 8.99 |  |  |  |  |  |  |
| 45 years old | 7.78 | 6.26 | 9.66 |  |  |  |  |  |  |
| 46 years old | 7.94 | 6.36 | 9.90 |  |  |  |  |  |  |
| 47 years old | 9.08 | 7.25 | 11.37 |  |  |  |  |  |  |
| 48 years old | 9.88 | 7.85 | 12.43 |  |  |  |  |  |  |
| 49 years old | 10.05 | 7.94 | 12.71 |  |  |  |  |  |  |
| 50 years old | 9.73 | 7.65 | 12.38 |  |  |  |  |  |  |
| 51 years old | 10.49 | 8.21 | 13.41 |  |  |  |  |  |  |
| 52 years old | 10.91 | 8.50 | 14.00 |  |  |  |  |  |  |
| 53 years old | 11.34 | 8.79 | 14.64 |  |  |  |  |  |  |
| 54 years old | 10.91 | 8.41 | 14.15 |  |  |  |  |  |  |
| 55 years old | 11.92 | 9.13 | 15.57 |  |  |  |  |  |  |
| 56 years old | 12.18 | 9.28 | 15.98 |  |  |  |  |  |  |
| 57 years old | 12.48 | 9.47 | 16.44 |  |  |  |  |  |  |
| 58 years old | 13.81 | 10.42 | 18.30 |  |  |  |  |  |  |
| 59 years old | 12.68 | 9.51 | 16.90 |  |  |  |  |  |  |
| 60 years old | 12.64 | 9.43 | 16.94 |  |  |  |  |  |  |
| 61 years old | 12.53 | 9.31 | 16.88 |  |  |  |  |  |  |
| 62 years old | 12.81 | 9.46 | 17.34 |  |  |  |  |  |  |
| 63 years old | 13.24 | 9.72 | 18.03 |  |  |  |  |  |  |
| 64 years old | 13.28 | 9.70 | 18.17 |  |  |  |  |  |  |
| 65 years old | 13.43 | 9.75 | 18.51 |  |  |  |  |  |  |
| 66 years old | 13.99 | 10.11 | 19.37 |  |  |  |  |  |  |
| 67 years old | 13.73 | 9.86 | 19.11 |  |  |  |  |  |  |
| 68 years old | 13.94 | 9.95 | 19.52 |  |  |  |  |  |  |
| 69 years old | 14.75 | 10.47 | 20.79 |  |  |  |  |  |  |
| 70 years old | 13.84 | 9.76 | 19.63 |  |  |  |  |  |  |
| 71 years old | 13.95 | 9.78 | 19.90 |  |  |  |  |  |  |
| 72 years old | 13.62 | 9.51 | 19.50 |  |  |  |  |  |  |
| 73 years old | 13.11 | 9.10 | 18.89 |  |  |  |  |  |  |
| 74 years old | 13.74 | 9.47 | 19.92 |  |  |  |  |  |  |
| 75 years old | 12.36 | 8.46 | 18.07 |  |  |  |  |  |  |
| 76 years old | 12.36 | 8.41 | 18.16 |  |  |  |  |  |  |
| 77 years old | 11.70 | 7.90 | 17.30 |  |  |  |  |  |  |
| 78 years old | 12.86 | 8.63 | 19.16 |  |  |  |  |  |  |
| 79 years old | 11.16 | 7.44 | 16.72 |  |  |  |  |  |  |
| 80 years old | 10.52 | 6.96 | 15.90 |  |  |  |  |  |  |
| 81 years old | 10.30 | 6.76 | 15.68 |  |  |  |  |  |  |
| 82 years old | 9.60 | 6.26 | 14.74 |  |  |  |  |  |  |
| 83 years old | 9.40 | 6.07 | 14.56 |  |  |  |  |  |  |
| 84 years old | 8.16 | 5.22 | 12.75 |  |  |  |  |  |  |
| 85 years old | 8.28 | 5.24 | 13.07 |  |  |  |  |  |  |

Note: 288,682 individual observations used in the analysis.

**Sensitivity analyses**

Our APC estimation results were robust to different grouping specifications. Age and period effects were very similar in the three sensitivity analyses. Only cohort effects differed from those obtained in Figure 3 when using 2-year age groups, 2-year survey periods, and 2-year birth cohorts. However, upper bounds estimates were closer to our results in Figure 3.

Figure S3: Sensitivity analysis 1: Estimated odds ratio (OR) and 95% confidence intervals of being a) overweight compared to healthy; and b) obese compared to healthy by 2-year groups of age, 2-year survey period and 2-year birth cohort using Health Survey England (1992-2019).

*a. Overweight compared to healthy BMI*

| 1. By 2-year age groups | 2. By 2-year survey period | 3. By 2-year birth cohort |
| --- | --- | --- |
| 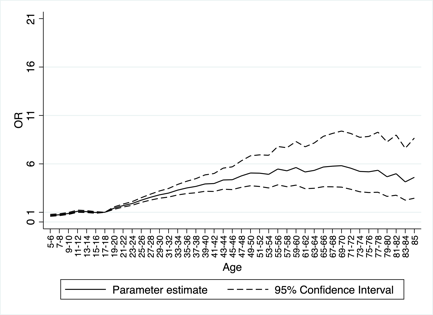 | 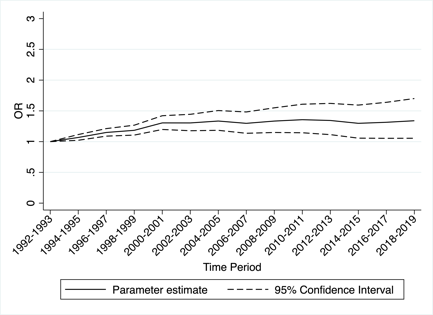 | 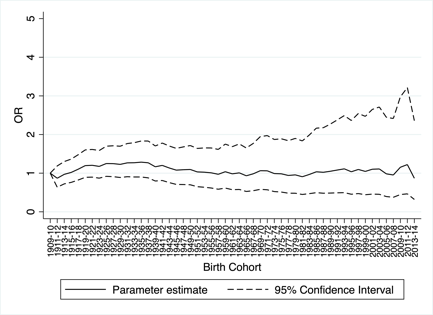 |

*b. Obese compared to healthy BMI*

| 1. By 2-year age groups | 2. By 2-year survey period | 3. By 2-year birth cohort |
| --- | --- | --- |
| 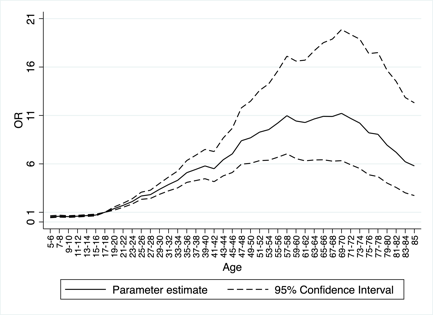 | 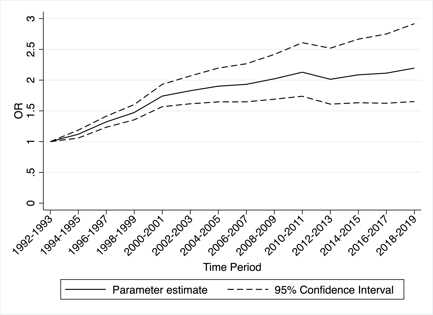 | 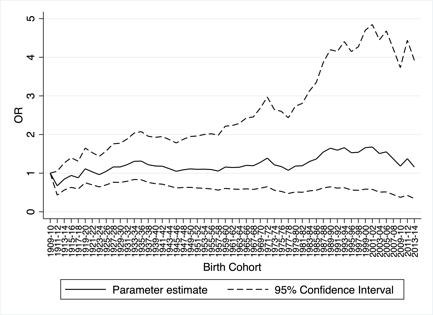 |

Note: Reference category for age is 17-18 years old, for period is the period 1992-1993, and for birth cohort is the birth cohort born between 1909-1910.

Figure S4: Sensitivity analysis 2: Estimated odds ratio (OR) and 95% confidence intervals of being a) overweight compared to healthy; and b) obese compared to healthy by 5-year groups of age, 5-year survey period and 5-year birth cohort using Health Survey England (1992-2019)

*a. Overweight compared to healthy BMI*

| 1. By 5-year age groups | 2. By 5-year survey period | 3. By 5-year birth cohort |
| --- | --- | --- |
| 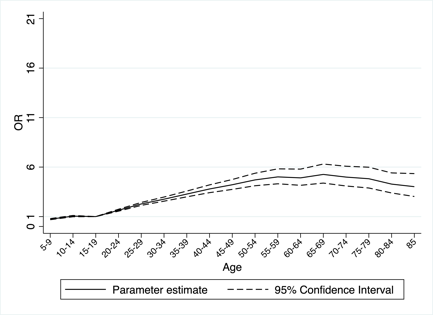 | 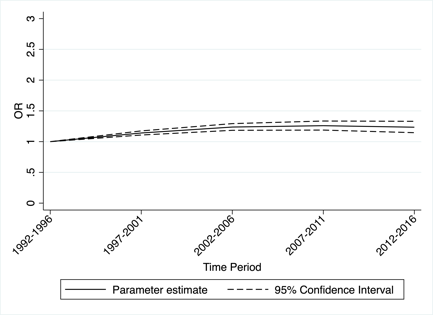 | 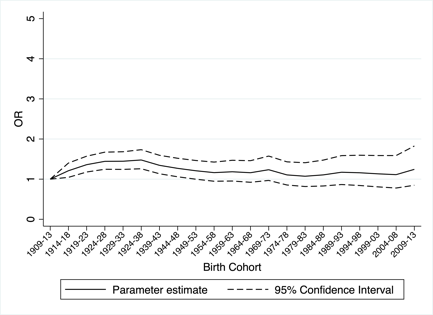 |

*b. Obese compared to healthy BMI*

| 1. By 5-year age groups | 2. By 5-year survey period | 3. By 5-year birth cohort |
| --- | --- | --- |
| 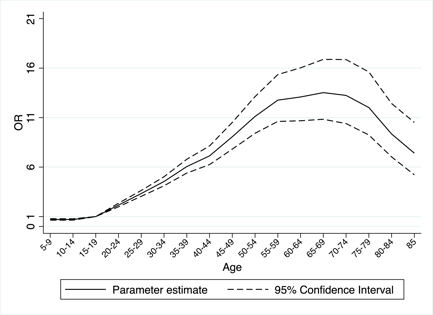 | 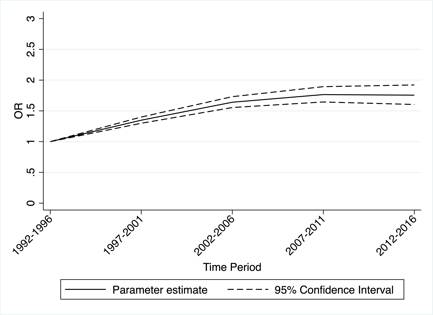 | 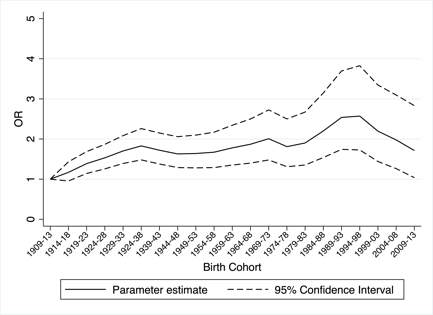 |

Figure S5: Sensitivity analysis 3: Estimated odds ratio (OR) and 95% confidence intervals of being a) overweight compared to healthy; and b) obese compared to healthy by single years of age, 2-year survey period and 10-year birth cohort using Health Survey England (1992-2019)

*a. Overweight compared to healthy BMI*

| 1. By single years of age | 2. By 2-year survey period | 3. By 10-year birth cohort |
| --- | --- | --- |
| 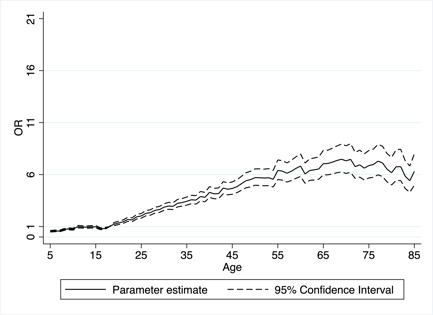 | 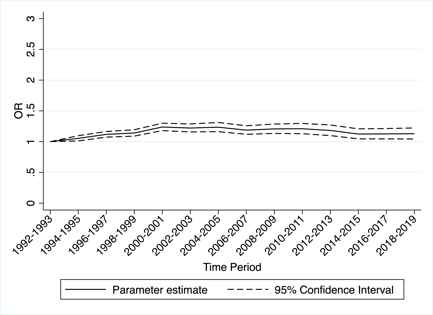 | 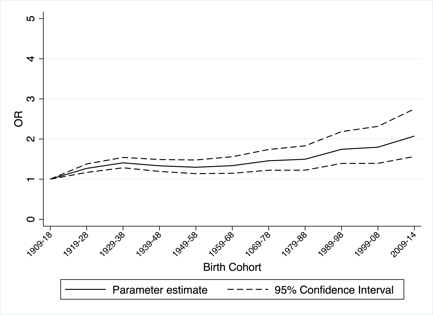 |

*b. Obese compared to healthy BMI*

| 1. By single years of age | 2. By 2-year survey period | 3. By 10-year birth cohort |
| --- | --- | --- |
| 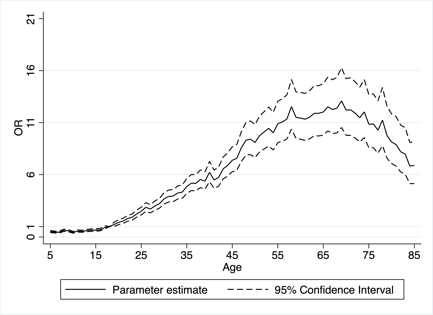 | 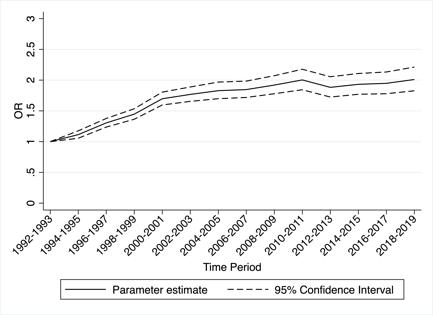 | 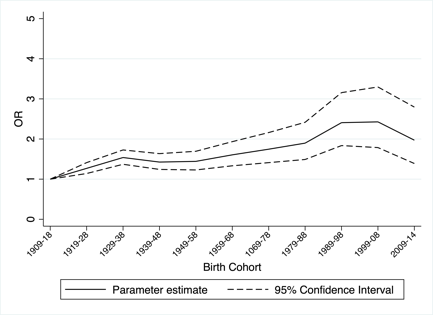 |

Note: Reference category for age is 18 years old, for period is the period 1992-1993, and for birth cohort is the birth cohort born between 1909-1918.

**References**

1. Office of Population Censuses and Surveys - Social Survey Division. Health Survey for England, 1991-1992 : Combined Data File. [data collection]. 1997. 2nd Edition. UK Data Service. SN: 3238.

2. Office of Population Censuses and Surveys - Social Survey Division. Health Survey for England, 1993. [data collection]. 1997. 2nd Edition. UK Data Service. SN: 3316.

3. Joint Health Surveys Unit of Social and Community Planning Research and University College London. Health Survey for England, 1994. [data collection]. 2017. 5th Edition. UK Data Service. SN: 3640.

4. Joint Health Surveys Unit of Social and Community Planning Research and University College London. Health Survey for England, 1995. [data collection]. 2017. 5th Edition. UK Data Service. SN: 3796.

5. Joint Health Surveys Unit of Social and Community Planning Research and University College London. Health Survey for England, 1996. [data collection]. 2017. 5th Edition. UK Data Service. SN: 3886.

6. Joint Health Surveys Unit of Social and Community Planning Research and University College London. Health Survey for England, 1997. [data collection]. 2017. 4th Edition. UK Data Service. SN: 3979.

7. University College London. Department of Epidemiology and Public Health. National Centre for Social Research. Health Survey for England, 1998. [data collection]. 2010. 5th Edition. UK Data Service. SN: 4150.

8. National Centre for Social Research. University College London. Department of Epidemiology and Public Health. Health Survey for England, 1999. [data collection]. 2010. 4th Edition. UK Data Service. SN: 4365.

9. University College London. Department of Epidemiology and Public Health. National Centre for Social Research. Health Survey for England, 2000. [data collection]. 2011. 4th Edition. UK Data Service. SN: 4487.

10. National Centre for Social Research. University College London. Department of Epidemiology and Public Health. Health Survey for England, 2001. [data collection]. 2010. 3rd Edition. UK Data Service. SN: 4628.

11. University College London. Department of Epidemiology and Public Health. National Centre for Social Research. Health Survey for England, 2002. [data collection]. 2010. 2nd Edition. UK Data Service. SN: 4912.

12. University College London. Department of Epidemiology and Public Health. National Centre for Social Research. Health Survey for England, 2003. [data collection]. 2010. 2nd Edition. UK Data Service. SN: 5098.

13. National Centre for Social Research. University College London. Department of Epidemiology and Public Health. Health Survey for England, 2004. [data collection]. 2010. 2nd Edition. UK Data Service. SN: 5439.

14. University College London. Department of Epidemiology and Public Health. National Centre for Social Research. Health Survey for England, 2005. [data collection]. 2010. 2nd Edition. UK Data Service. SN: 5675.

15. National Centre for Social Research. University College London. Department of Epidemiology and Public Health. Health Survey for England, 2006. [data collection]. 2010. 4th Edition. UK Data Service. SN: 5809.

16. National Centre for Social Research. University College London. Department of Epidemiology and Public Health. Health Survey for England, 2007. [data collection]. 2010. 2nd Edition. UK Data Service. SN: 6112.

17. National Centre for Social Research. University College London. Department of Epidemiology and Public Health. Health Survey for England, 2008. [data collection]. 2013. 4th Edition. UK Data Service. SN: 6397.

18. University College London. Department of Epidemiology and Public Health. National Centre for Social Research. Health Survey for England, 2009. [data collection]. 2015. 3rd Edition. UK Data Service. SN: 6732.

19. NatCen Social Research. Royal Free and University College Medical School. Department of Epidemiology and Public Health. Health Survey for England, 2010. [data collection]. 2015. 3rd Edition. UK Data Service. SN: 6986.

20. University College London. Department of Epidemiology and Public Health. NatCentre Social Research. Health Survey for England, 2011. [data collection]. 2013. UK Data Service. SN: 7260.

21. NatCen Social Research. University College London. Department of Epidemiology and Public Health. Health Survey for England, 2012. [data collection]. 2014. UK Data Service. SN: 7480.

22. NatCen Social Research. University College London. Department of Epidemiology and Public Health. Health Survey for England, 2013. [data collection]. 2015. UK Data Service. SN: 7649.

23. NatCen Social Research. University College London. Department of Epidemiology and Public Health. Health Survey for England, 2014. [data collection]. 2018. 3rd Edition. UK Data Service. SN: 7919.

24. NatCen Social Research. University College London. Department of Epidemiology and Public Health. Health Survey for England, 2015. [data collection]. 2019. 2nd Edition. UK Data Service. SN: 8280.

25. NatCen Social Research. University College London. Department of Epidemiology and Public Health. Health Survey for England, 2016. [data collection]. 2019. 3rd Edition. UK Data Service. SN: 8334.

26. University College London. Department of Epidemiology and Public Health. National Centre for Social Research (NatCen). Health Survey for England, 2017. [data collection]. 2020. 2nd Edition. UK Data Service. SN: 8488.

27. National Centre for Social Research (NatCen). University College London. Department of Epidemiology and Public Health. Health Survey for England, 2018. [data collection]. 2020. UK Data Service. SN: 8649.

28. National Centre for Social Research (NatCen). University College London. Department of Epidemiology and Public Health. Health Survey for England, 2019. [data collection]. 2021. UK Data Service. SN: 8860.

29. NatCen Social Research and UCL, Health Survey for England 2015 Methods. 2016: <http://healthsurvey.hscic.gov.uk/media/37742/hse2015-methods.pdf>. [Accessed 28 February 2022].

30. Gillespie, D., et al., *Health Survey Data Wrangling*. 2022: <https://stapm.gitlab.io/r-packages/hseclean/index.html>.

31. Opazo Breton, M., et al., *Understanding long-term trends in smoking in England, 1972-2019: an age-period-cohort approach.* Addiction, 2021. **n/a**(n/a). DOI: <https://doi.org/10.1111/add.15696>.

32. Wang, Z., et al., *Predicting age by mining electronic medical records with deep learning characterizes differences between chronological and physiological age.* Journal of Biomedical Informatics, 2017. **76**: p. 59-68. DOI: <https://doi.org/10.1016/j.jbi.2017.11.003>.

33. Jylhävä, J., N.L. Pedersen, and S. Hägg, *Biological Age Predictors.* EBioMedicine, 2017. **21**: p. 29-36. DOI: <https://doi.org/10.1016/j.ebiom.2017.03.046>.
